# Changes of esophageal varices in hepatitis C patients after achievement of a sustained viral response by direct‐acting antivirals

**DOI:** 10.1002/deo2.11

**Published:** 2021-07-05

**Authors:** Satoshi Takakusagi, Naoto Saito, Takashi Ueno, Takeshi Hatanaka, Masashi Namikawa, Hiroki Tojima, Daichi Takizawa, Atsushi Naganuma, Takashi Kosone, Hirotaka Arai, Ken Sato, Satoru Kakizaki, Hitoshi Takagi, Toshio Uraoka

**Affiliations:** ^1^ Department of Gastroenterology and Hepatology Kusunoki Hospital Gunma Japan; ^2^ Department of Gastroenterology Gunma Saiseikai Maebashi Hospital Gunma Japan; ^3^ Department of Internal Medicine Isesaki Municipal Hospital Gunma Japan; ^4^ Department of Internal Medicine Kiryu Kosei General Hospital Gunma Japan; ^5^ Department of Gastroenterology and Hepatology Gunma University Graduate School of Medicine Gunma Japan; ^6^ Department of Gastroenterology Japanese Red Cross Maebashi Hospital Gunma Japan; ^7^ Department of Gastroenterology National Hospital Organization Takasaki General Medical Center Gunma Japan; ^8^ Department of Clinical Research National Hospital Organization Takasaki General Medical Center Gunma Japan

**Keywords:** direct acting antivirals, esophageal varices, FIB‐4, hepatitis C, sustained viral response

## Abstract

**Objectives:**

The changes in portal hypertension after achieving a sustained viral response (SVR) by direct‐acting antivirals (DAAs) have not been fully elucidated. Consequently, noninvasive and inexpensive predictors need to be investigated. We therefore explored factors associated with the progression of EVs after the achievement of an SVR with DAAs in patients with chronic hepatitis C.

**Methods:**

Eighty‐nine patients, who had achieved an SVR with DAAs and could have their esophagogastroduodenoscopy (EGD) findings compared between before DAAs administration and after achieving an SVR achievement were enrolled in this study. We compared the patients with and without EVs progression. Furthermore, the cumulative progression rates of EVs were also analyzed.

**Results:**

The fibrosis‐4 index (FIB‐4) before DAAs administration was the only significant factor for the progression of EVs after an SVR (odds ratios: 1.2, 95% confidence intervals: 1.05–1.38, *p* = 0.01). In a receiver operating characteristics analysis, the cut‐off of FIB‐4 for the progression of EVs was 8.41 (sensitivity: 0.63, specificity: 0.86, positive predictive value: 0.31, negative predictive value: 0.96), namely EVs of those with more than 8.41 of FIB‐4 progressed and those with less than 8.41 of FIB‐4 did not.

**Conclusions:**

As patients with FIB‐4 ≥ 8.41 may have progressions of EVs, periodic surveillance by EGD should be continued in such cases, even after an SVR is achieved.

## INTRODUCTION

Direct‐acting antivirals (DAAs) have become the first‐line treatment for patients with chronic hepatitis C (CHC) worldwide. In Japan, glecaprevir/pibrentasvir (GLE/PIB) is now most prevalently used for the treatment of CHC, with high hepatitis C virus (HCV) eradication rates (>95%) among treatment‐naïve patients.^1^, ^2^ Furthermore, the introduction of sofosbuvir/velpatasvir (SOF/VEL) is expected to be highly effective in patients with decompensated cirrhosis.^3^ The combination therapy of SOF/VEL and ribavirin was reported to be effective even in patients who experienced virologic failure after previous DAAs treatment.^4^

The hepatic venous pressure gradient (HVPG) has been considered a typical surrogate marker for portal pressure.^5^ Patients with CHC and compensated or decompensated cirrhosis who achieved a sustained viral response (SVR) were reportedly able to achieve a clinically meaningful reduction in the HVPG at the long‐term follow‐up.^6^ However, it was also reported that clinically significant portal hypertension (CSPH) persisted in most patients, even after achieving an SVR,^7^, ^8^ resulting in a continuous risk of decompensation and death.^8^ Gastrointestinal hemorrhaging due to ruptured esophageal varices (EVs) is a significant cause of mortality in patients with HCV‐related cirrhosis.^9^ The Baveno VI guidelines recommend avoiding performing endoscopies for surveillance of EVs in patients with liver stiffness measurements of <20 kPa and platelet counts > 150 × 10^9^/L,^10^ and the expanded‐Baveno VI guidelines also recommend avoiding performing endoscopies in patients with liver stiffness measurements < 25 kPa and platelet counts > 110 × 10^9^/L.^11^ However, as transient elastography is expensive, it is not available in many facilities. The factors associated with the progression of EVs after achieving an SVR with DAAs remain unclear, and noninvasive and inexpensive predictors of EVs have not been established. Then, we retrospectively compared the endoscopic findings before DAAs administration with those after achieving an SVR and clarified the factors associated with the progression and improvement of EVs after achieving an SVR.

## PATIENTS AND METHODS

### Patients

**FIGURE 1:**
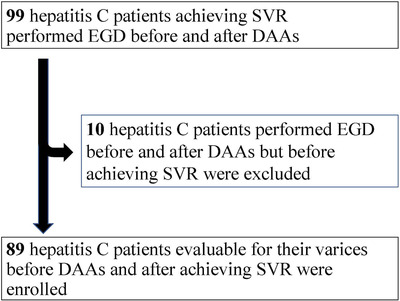
The flow‐chart of patient selection of this study. DAAs, direct‐acting antivirals; EGD, esophagogastroduodenoscopy; SVR, sustained viral response

In 99 patients who achieved SVR at post‐treatment week 24 (SVR24) by October 2020 with DAAs (daclatasvir+asunaprevir, elbasvir+grazoprevir, GLE/PIB, sofosbuvir/ledipasvir [SOF/LDV], sofosbuvir+ribavirin, or SOF/VEL) at seven affiliated hospitals, 89 patients could have endoscopic findings compared before and after DAAs and were potentially eligible for the study (**Figure** **1**). Ten patients, whose endoscopic findings after DAAs had been evaluated before SVR24, were excluded. All enrolled patients had never been treated for gastric varices and/or EVs before DAAs administration. None of them were complicated with hepatitis B virus and/or human immunodeficiency virus infection, portal vein thrombosis including the invasion of hepatocellular carcinoma, alcoholic cirrhosis, liver cirrhosis associated with non‐alcoholic steatohepatitis, autoimmune hepatitis, primary biliary cholangitis, primary sclerosing cholangitis, congestive cirrhosis, hemochromatosis, and Wilson's disease.

### Surveillance of EVs and the study endpoint

EVs were morphologically classified into four groups (none, F1, F2, and F3) by experienced endoscopists based on the current Japanese guideline.^12^ In principle, EVs were assessed every 6–12 months on esophagogastroduodenoscopy (EGD). Progression of EVs was endoscopically defined as the appearance of new EVs, enlargement of existing EVs, appearance of new red color sign in existing EVs, and rupture of EVs. Furthermore, improvement of EVs was also defined as a reduction in existing EVs.

We retrospectively investigated the factors associated with the progression and improvement of EVs after achieving an SVR24. The observation period was defined as the interval from the date of completion of DAAs administration to the first confirmation of EVs progression on EGD or the last follow‐up EGD.

### Demographic and laboratory data

The demographic data, such as the sex, age, interval from the latest EGD before DAAs administration to the start of DAAs, DAAs used, and laboratory data at the initiation of DAAs administration, such as the platelet count, alanine aminotransferase (ALT), aspartate aminotransferase (AST), and HCV‐RNA levels, were included as baseline data. The fibrosis‐4 index (FIB‐4) has been widely used as a liver fibrosis marker^13^ which is calculated by the following formula: (age × AST) / (platelet count × ALT^1/2^).

### Virologic assessments

An SVR24 was defined as continuously undetectable serum HCV‐RNA on a polymerase chain reaction assay until 24 weeks after the end of DAA administration.

### Ethical considerations

This study was approved by the institutional review board at each affiliated hospital, and the need for written informed consent was waived because of the retrospective nature of the study. In addition, this study complied with the Ethical Guidelines for Medical and Health Research Involving Human Subjects in Japan.

### Statistical analyses

Continuous variables were expressed as the median (interquartile range [IQR]) and analyzed by the Mann‐Whitney U test as nonparametric and unpaired analyses. Categorical variables were analyzed by Fisher's exact test. The cut‐off of continuous variable was determined by a receiver operating characteristic (ROC) analysis. The cumulative incidence curve was determined, and the differences among groups were assessed using Gray's test. A multivariate analysis was performed by a logistic regression analysis. All statistical analyses were performed using EZR version 1.53 (Saitama Medical Center, Jichi Medical University, Saitama, Japan).^14^

## RESULTS

### Baseline characteristics of the study patients before the treatment with DAAs

The interval from the latest EGD before DAAs administration to the start of DAAs administration was 221 days (95–375) (**Table** **1**). The study included 41 males and 48 females with a median age of 71 years old (66–76). Forty‐one patients (46.1%) were diagnosed with liver cirrhosis due to a platelet count < 100 × 10^9^/L or the presence of EVs. Among those, two patients, who had been treated with SOF/VEL, were judged to have Child‐Pugh B decompensated cirrhosis. Regarding the grade of EVs, 61 patients (68.5%) had no EVs, while F1 and F2 EVs were endoscopically confirmed in 26 patients (29.2%) and two patients (2.2%) before the start of DAA administration, respectively. F3 EVs and EVs with red color sign were not detected in any patients.

**TABLE 1:** Baseline characteristics of the study patients before the treatment with direct‐acting antivirals (DAAs)

Variables	All cases (*n* = 89)
Age (years)	71 (66–76)
Male, *n* (%)	41 (46.1)
Platelet count (×10^9^/L)	118 (90–153)
AST (IU/L)	47 (37–67)
ALT (IU/L)	38 (30–54)
FIB‐4	4.61 (3.33–7.01)
HCV‐RNA (log IU/ml)	6.1 (5.6–6.4)
Liver cirrhosis, *n* (%)	41 (46.1)
EVs, none/F1/F2/F3, *n* (%)	61 (68.5)/26 (29.2)/2 (2.2)/0 (0)
Interval from the latest EGD before DAAs to the start of DAAs (days)	221 (95–375)
DAAs regimen, *n* (%)	
Daclatasvir/Asunaprevir	38 (42.7)
Elbasvir/Grazoprevir	2 (2.2)
Pibrentasvir/Glecaprevir	5 (5.6)
Sofosbuvir/Ledipasvir	29 (32.6)
Sofosbuvir/Ribavirin	13 (14.6)
Sofosbuvir/Velpatasvir	2 (2.2)

Abbreviations: ALT, alanine aminotransferase; AST, aspartate aminotransferase; DAAs, direct‐acting antiviral agents; EVs, esophageal varices; FIB‐4, fibrosis‐4; HCV, hepatitis C virus.

### Factors associated with progression of EVs

**FIGURE 2:**
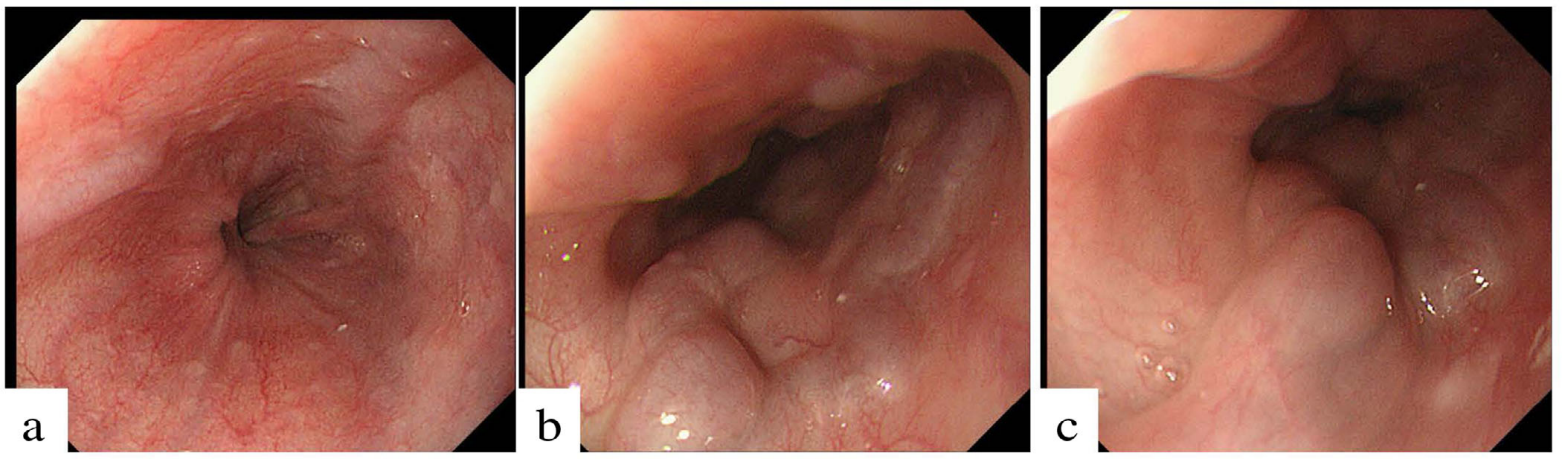
The findings of esophagogastroduodenoscopy before and after direct‐acting antivirals (DAAs) in the example case 1. A 70‐year‐old female was confirmed to have had F1 esophageal varices (EVs) before DAA administration (**a**). Her FIB‐4 before DAAs administration was 12.07. Six hundred twenty‐four days after treatment with SOF/LDV, the EVs had progressed to F2 (**b and c**). The patient was then treated by endoscopic injection sclerotherapy

**FIGURE 3:**
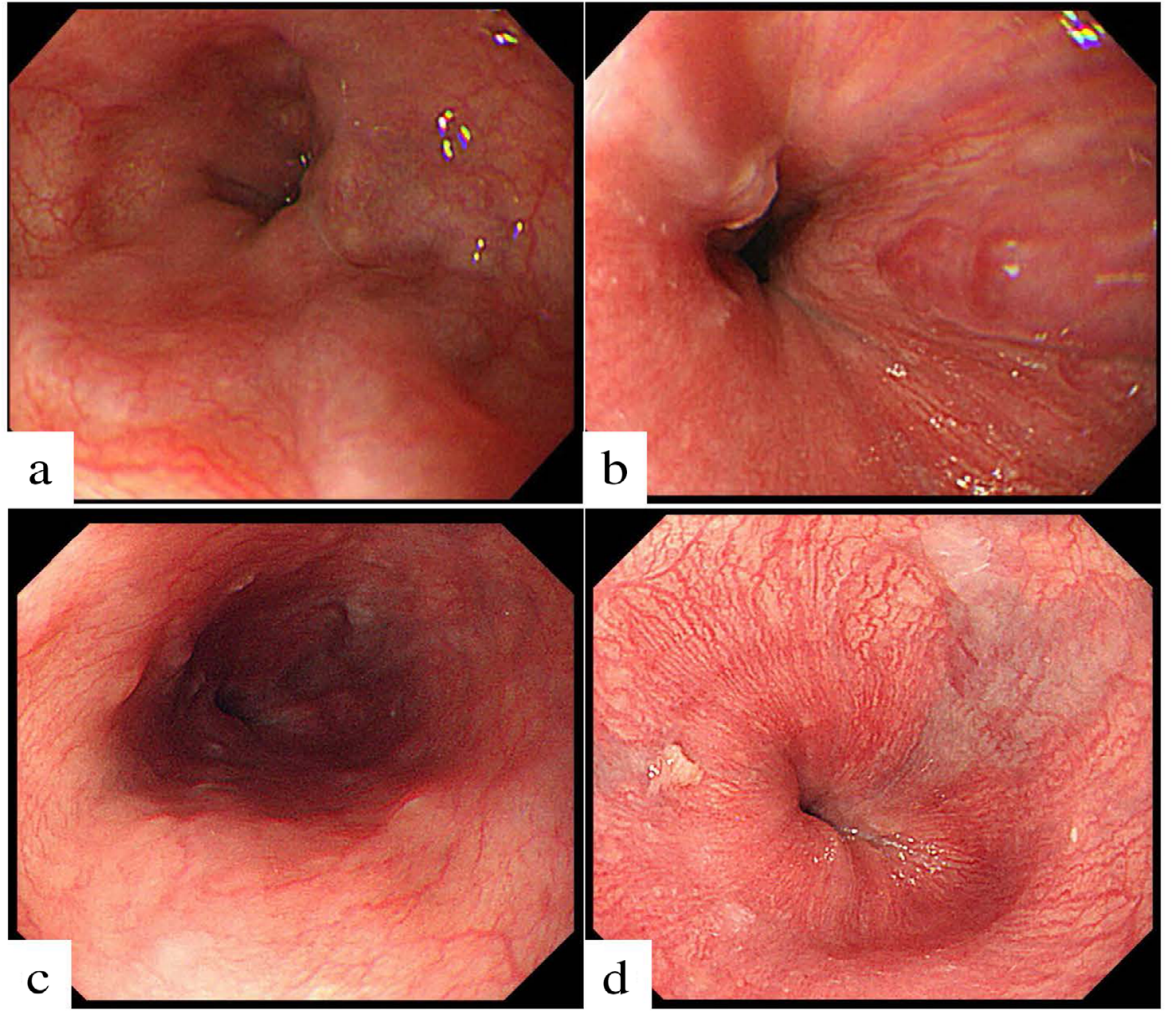
The findings of esophagogastroduodenoscopy before and after direct‐acting antivirals (DAAs) in the example case 2. A 69‐year‐old female was confirmed to have had F2 esophageal varices (EVs) before DAAs administration (**a and b**). Her FIB‐4 before DAAs administration was 3.54. Five hundred forty‐nine days after treatment with SOV/LDV, EVs were improved to be a small amount of F1 adjacent to esophagogastric junction (**c and d**)

**FIGURE 4:**
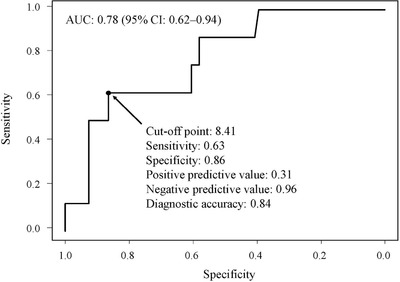
A receiver operating characteristics analysis regarding the cut‐off of FIB‐4 for the progression of esophageal varices after achieving a sustained viral response. According to the Youden index, the best cut‐off of FIB‐4 was 8.41 (sensitivity: 0.63, specificity: 0.86, positive predictive value: 0.31, negative predictive value: 0.96, diagnostic accuracy: 0.84). The area under the curve was 0.78 (95% confidence interval: 0.62–0.94)

During the observation period, 76 patients (85.4%) had no change of EVs, eight patients (9.0%) progressed like the example case 1 (**Table** **2**) (**Figure** **2**), and five patients (5.6%) improved like the example case 2 (**Figure** **3**). No varices rupture was reported. In a univariate analysis, only the FIB‐4 was a significant predictive factor for the EVs progression (*p* = 0.01). No significant differences were demonstrated in the observation period, age, sex, AST, ALT, HCV‐RNA levels, platelet counts, the rates of liver cirrhosis, the grade of EVs, and the regimen of DAAs between the patients with and without progression of EVs. Even in the multivariate analysis, the FIB‐4 was a statistically significant predictive factor for the progression of EVs after achieving an SVR (odds ratios: 1.2 [95% confidence intervals: 1.05–‐1.38], *p* = 0.01). In the ROC analysis, the cut‐off of the FIB‐4 for the progression of EVs was 8.41 (sensitivity: 0.63, specificity: 0.86, positive predictive value: 0.31, negative predictive value: 0.96, diagnostic accuracy: 0.84). The area under the curve was 0.78 (95% confidence interval: 0.62–‐0.94) (**Figure** **4**). The backgrounds of eight patients, who had the progression of EVs after SVR, are shown in **Table** **3**.

**TABLE 2:** The factors associated with esophageal varices (EVs) progression after SVR (*n* = 89)

			Univariate analysis	Multivariate analysis	
	EVs progression (‐) (*n* = 81)	EVs progression (+) (*n* = 8)	*p* value	Odds ratio (95% confidence interval)	*p* value
Age (years)	71 (66–76)	71 (67–78)	0.40		
Male, *n* (%)	37 (45.7)	4 (50)	0.90		
Platelet count (×10^9^/L)	120 (92–157)	94 (61–125)	0.08		
AST (IU/L)	46 (36–66)	48 (40–69)	0.08		
ALT (IU/L)	38 (29–53)	48 (40–69)	0.12		
FIB‐4	4.48 (3.25–6.67)	9.94 (5.2–11.9)	0.01	1.2 (1.05–1.38)	0.01
HCV‐RNA (log IU/ml)	6.1 (5.6–6.4)	6.2 (5.9–6.4)	0.40		
Liver cirrhosis, *n* (%)	36 (44.4)	5 (62.5)	0.46		
Grade of EVs, none/F1/F2/F3, *n* (%)	56 (69.1)/23 (28.4)/2 (2.5)/0 (0)	5 (62.5)/3 (37.5)/0 (0)/0 (0)	0.74		
Observation period (days)	688 (407–1090)	609.5 (423.8–773)	0.74		
DAAs regimen, *n* (%)					
Daclatasvir/Asunaprevir	34 (42)	4 (50)	0.73		
Elbasvir/Grazoprevir	2 (2.5)	0 (0)			
Pibrentasvir/Glecaprevir	5 (6.2)	0 (0)			
Sofosbuvir/Ledipasvir	25 (30.9)	4 (50)			
Sofosbuvir/Ribavirin	13 (16.0)	0 (0)			
Sofosbuvir/Velpatasvir	2 (2.5)	0 (0)			

Abbreviations: ALT, alanine aminotransferase; AST, aspartate aminotransferase; DAAs, direct‐acting antiviral agents; EVs, esophageal varices; FIB‐4, fibrosis‐4; HCV, hepatitis C virus; SVR, sustained viral response.

**TABLE 3:** The backgrounds of the cases with the progression of esophageal varices (EVs) after sustained viral response (SVR)

	Case 1	Case 2	Case 3	Case 4	Case 5	Case 6	Case 7	Case 8
Age (years)	67	77	81	86	64	68	70	73
Sex	Male	Male	Female	Female	Female	Male	Female	Male
Platelet count (×10^9^/L)	34	173	130	69	79	109	123	37
AST (IU/L)	102	55	54	67	84	43	269	37
ALT (IU/L)	78	24	41	53	66	42	162	38
FIB‐4	22.8	5	5.27	11.47	8.41	4.13	12.07	11.84
HCV‐RNA (log IU/ml)	6.8	5.6	6.1	6.0	5.7	6.2	6.9	6.3
DAAs regimen	D+A	D+A	D+A	D+A	S/L	S/L	S/L	S/L
EVs before DAAs								
F	(−)	(−)	(−)	F1	(−)	(−)	F1	F1
RCS	(−)	(−)	(−)	(−)	(−)	(−)	(−)	(−)
EVs after SVR								
F	F1	F2	F1	F2	F2	F2	F2	F2
RCS	(−)	(+)	(−)	(−)	(−)	(+)	(−)	(−)
Observation period (days)	451	1181	194	595	637	1626	624	342

Abbreviations: ALT, alanine aminotransferase; AST, aspartate aminotransferase; CH, chronic hepatitis; DAAs, direct‐acting antiviral agents; D+A, daclatasvir+asunaprevir; EVs, esophageal varices; FIB‐4, fibrosis‐4; HCV, hepatitis C virus; LC, liver cirrhosis; RCS, red color sign; SVR, sustained viral response; S/L, sofosbuvir/ledipasvir.

### Cumulative EVs progression rates

**FIGURE 5:**
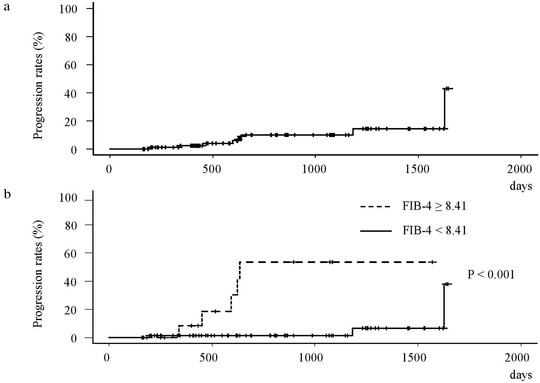
**(a**) The cumulative progression rates of esophageal varices (EVs) after achieving a sustained viral response in overall patients. (**b)** The cumulative progression rates of EVs classified by the cut‐off value of the FIB‐4. The cumulative progression rates of EVs at 1, 2, 3, and 4 years were 2.6%, 10.0%, 10.0%, and 14.5%, respectively (**a**). A significant difference in the cumulative EVs progression rates was demonstrated between the patients with FIB‐4 ≥ 8.41 and those with FIB‐4 < 8.41 (*p* <0.001) (**b**)

The median observation period (IQR) for the progression of EVs in the overall patients of this study was 637 days (407–‐1090). The cumulative EVs progression rates at 1, 2, 3, and 4 years during that period were 2.6%, 10.0%, 10.0%, and 14.5%, respectively (**Figure** **5a**). In the patients with FIB‐4 ≥ 8.41, the cumulative EVs progression rates at 1, 2, 3, and 4 years were 8.3%, 53.4%, 53.4%, and 53.4%, respectively. Furthermore, in the patients with FIB‐4 < 8.41, the cumulative EVs progression rates at 1, 2, 3, and 4 years were 1.4%, 1.4%, 1.4%, and 6.6%, respectively. A significant difference in the cumulative EVs progression rates was demonstrated between the patients with an FIB‐4 ≥ 8.41 and those with an FIB‐4 < 8.41 (P < 0.001) (**Figure** **5b**).

### Factors associated with improvement of EVs

The factors associated with the improvement of EVs were analyzed in 28 patients who were confirmed to have had EVs before DAAs administration. As already mentioned, during the observation period, the improvement of EVs was observed in five patients (17.9% of analyzed 28 patients). However, no significant differences in the observation period, age, sex, AST, ALT, HCV‐RNA levels, FIB‐4, platelet counts, grade of EVs, or DAAs regimen were observed between the patients with and without improvement of EVs (**Table** **4**).

**TABLE 4:** The factors associated with esophageal varices (EVs) improvement after sustained viral response (SVR) (*n* = 28)

	EVs improvement (−) (*n* = 23)	EVs improvement (+) (*n* = 5)	*p* value
Age (years)	71 (67–76)	74 (69–75)	0.98
Male, *n* (%)	9 (39.1)	1 (20)	0.63
Platelet count (×10^9^/L)	90 (67.5–113)	103 (62–138)	0.81
AST (IU/L)	56 (41–81)	49 (46–50)	0.95
ALT (IU/L)	42 (36–54.5)	35 (31–50)	0.32
FIB‐4	7.1 (4.64–10.87)	7.93 (4.48–9.69)	0.88
HCV‐RNA (log IU/ml)	5.6 (5.1–6.2)	6.0 (6.0–6.2)	0.55
Grade of EVs, F1/F2/F3, *n* (%)	22 (95.7)/1 (4.3)/0 (0)	4 (80)/1 (20)/0 (0)	0.33
Observation period (days)	437 (337–644)	1231 (549–1270)	0.09
DAAs regimen, *n* (%)			
Daclatasvir/Asunaprevir	5 (21.7)	2 (40)	0.44
Elbasvir/Grazoprevir	1 (4.3)	0 (0)	
Pibrentasvir/Glecaprevir	1 (4.3)	1 (20)	
Sofosbuvir/Ledipasvir	12 (52.2)	1 (20)	
Sofosbuvir/Ribavirin	2 (8.7)	1 (20)	
Sofosbuvir/Velpatasvir	2 (8.7)	0 (0)	

All of the patients analyzed in this table had EV before DAAs therapy.

Abbreviations: ALT, alanine aminotransferase; AST, aspartate aminotransferase; DAAs, direct‐acting antiviral agents; EVs, esophageal varices; FIB‐4, fibrosis‐4; HCV, hepatitis C virus; SVR, sustained viral response.

### Anti‐portal hypertensive drugs

One patient had been received angiotensin receptor blocker (ARB) and other two patients had been received beta‐blocker in five patients with improvement of EVs during the observation period. On the other hand, only one patient EVs had been received ARB in eight patients with the progression of EVs. All of these patients were administered these drugs not for portal hypertension but for hypertension.

## DISCUSSION

This study investigated the progression of EVs after achieving an SVR with DAAs and demonstrated that the cut‐off value of FIB‐4 before DAAs administration had an excellent negative predictive value for EVs progression. The frequency of endoscopic screening for EVs can be reduced in patients with an FIB‐4 < 8.41 before DAAs administration.

Yuri et al reported that the 1‐ and 3‐year cumulative gastroesophageal varices (GEVs) progression rates after achieving an SVR with DAAs were 8.2% and 32.3%, respectively.^15^ Although those rates seemed to be higher than those in our study, this might have been due to differences in the background characteristics of the subjects. Namely, all 37 patients in that previous report had had liver cirrhosis with GEVs since before DAAs administration, and among them, 15 (40.5%) had had decompensated cirrhosis of Child‐Pugh class B. In contrast, in our study, although 41 patients (46.1%) had been diagnosed with liver cirrhosis, only 28 (31.5%) had already been diagnosed with EVs before DAAs administration. Furthermore, there were only two patients who had already been diagnosed as Child‐Pugh class B, and there were no cases of Child‐Pugh class C in our study. Based on the above findings, it was considered that the small number of patients with advanced liver fibrosis was responsible for the relatively low cumulative EVs progression rates in our study. Although non‐SVR cases were too few to compare because of excellent efficacy of DAAs to eradicate HCV and could not be set as a control group in this study, the previous study reported that the 1‐, 3‐, 5‐, and 7‐year cumulative progression rates of GEVs in non‐SVR cases were 9.2%, 33.7%, 45.4%, and 78.5%.^15^ These cumulative progression rates seemed to considerably higher than those of our study even with consideration of the differences in the backgrounds of the subjects, suggesting the efficacy of SVR with DAAs for suppressing EVs progression.

It was also reported that the proportion of GEVs progression in patients with no or F1 EVs was significantly lower than that in patients with F2 or F3 EVs.^15^ In our study, although the number of patients with F2 EVs before DAAs administration was small, the progression of EVs after achieving an SVR was observed in five of 61 patients (8.2%) without EVs before DAAs administration. Furthermore, a decrease in EVs after achieving an SVR was observed in one of two patients (50%) with F2 EVs before DAAs administration. A recent study reported that among 64 patients without baseline GEVs, eight (12.5%) developed GEVs after achieving an SVR, and among 50 patients with baseline low‐risk GEVs (<5 mm), 12 patients (24%) developed high‐risk GEVs after achieving an SVR.^16^ Given these findings, it seems difficult to simply predict progression of EVs and CSPH after achieving an SVR based solely on the endoscopic findings before DAAs administration.

The FIB‐4 was the only significant factor predicting the progression of EVs after achieving an SVR and EVs may progress in cases with an FIB‐4 ≥ 8.41. In contrast, most cases with an FIB‐4 < 8.41 before DAAs administration did not show progression of EVs after achieving an SVR. Given these findings, the cut‐off of 8.41 for the FIB‐4 was considered useful for excluding cases with progression of EVs after achieving an SVR. Thus far, EGD has remained the gold standard for detecting EVs, and the recent Baveno VI Meeting Consensus recommended EGD surveillance for all cirrhotic patients at the time of their diagnosis and periodic endoscopy examination in patients with EVs.^10^ However, routine EGD surveillance may not be cost‐effective because less than 50% of patients with liver cirrhosis have EVs.^17^ The FIB‐4 has been established as a noninvasive marker of liver fibrosis determined based on routine laboratory parameters. It has been reported that the FIB‐4 > 3.25 had the positive predictive value of 82.1% to confirm the existence of significant liver fibrosis with the specificity of 98.2%.^13^ From these findings, the determination of the surveillance frequency of EVs by EGD after achieving an SVR using the FIB‐4 was reasonable in terms of both cost‐effectiveness and reduced discomfort of EGD.

Since endoscopic examinations and treatments have been recognized as useful modalities for preventing bleeding death in patients with EVs, EGD tended to be performed more frequently than the above criteria recommended. Kraja et al reported that the FIB‐4 was useful for predicting the presence of EVs but not the rupture of EVs.^18^ However, according to our results, the progression of EVs after achieving an SVR was rare in the patients with an FIB‐4 < 8.41 before DAAs administration, so the frequency of EGD surveillance may be able to be reduced, especially in patients without EVs or with F1 EVs. However, CSPH may persist with EVs progression even after achieving an SVR in patients with FIB‐4 ≥ 8.41, so careful EGD surveillance should still be continued in these cases.

Regarding the improvement of EVs, five of 28 patients with confirmation of EVs before DAAs administration showed improvement of EVs after achieving an SVR with DAAs. However, no significant factors associated with the overall improvement of EVs were identified in our study, including the FIB‐4. On the other hand, three of five patients with the improvements of EVs and one of eight patients with EVs progression after SVR had been coincidentally received ARB or beta‐blocker for the diseases other than portal hypertension. Although the efficacy of these medicines for portal hypertension has already been reported,^10^, ^19^ more subjects under prospective randomized study would be mandatory for the evaluation of EVs in HCV‐SVR patients. These medicines may be a useful option to prevent progression of EVs after SVR in patients with mild EVs before DAAs. However, as risky varices, such as F2, F3 EVs, and EVs with a red color sign, are known to demonstrate high‐risk of bleeding,^12^, ^20^ prophylactic treatments including endoscopic variceal ligation and endoscopic injection sclerotherapy should be performed before antiviral treatment with DAAs, regardless of the FIB‐4 in such cases.

In our study, most enrolled patients had a good liver functional reserve without EVs or with mild EVs. However, even in such subjects, since EVs had significantly progressed in the patients with high FIB‐4 levels, there might be ‘'a point of no return'’ in which HCV eradication was no longer able to suppress the progression of CSPH might, even in Child‐Pugh class A without EVs or with only mild EVs. Regarding patients with decompensated cirrhosis, those with Child‐Pugh class B were less likely to have a decreased HVPG due to achieving an SVR than those with Child‐Pugh class A,^21^ CSPH can persist in patients with decompensated cirrhosis of Child‐Pugh class B or C, even after achieving an SVR, so careful endoscopic surveillance for EVs should be continued in such cases.

The present study has several limitations, such as its retrospective cohort nature, relatively small number of severe EVs subjects, a lack of control groups such as non‐SVR cases, and short observation period. Further, the relative long duration between the latest endoscopy and DAAs treatment might have caused both overestimation for the progression of EVs and underestimation for improvement of those. As such, a further large‐scale, prospective study will be needed.

In conclusion, the FIB‐4 was useful as a noninvasive and inexpensive predicter of the progression of EVs after achieving an SVR by treatment with DAAs. The progression of EVs was observed even after achieving an SVR in patients with FIB‐4 ≥ 8.41. Therefore, these cases should be periodically surveyed by EGD for their EVs.

## CONFLICT OF INTEREST

Ken Sato received research funding from AbbVie Inc. Satoru Kakizaki received lecture fees from AbbVie Inc. and Gilead Sciences Inc. Author T.U. is DEIC of *DEN Open*.

## FUNDING INFORMATION

None.
